# Acute Myeloid Leukemia, Myelodysplasia‐Related (AML‐MR), With del(5q) and Double Minutes Containing Chromosomal Segment 11q24 Leading to Amplification and Expression of FLI1

**DOI:** 10.1155/crh/2250750

**Published:** 2026-04-13

**Authors:** Satomi Hiya, Joseph Tripodi, John Mascarenhas, Alina Dulau Florea, Vesna Najfeld

**Affiliations:** ^1^ Department of Pathology, Molecular, and Cell-Based Medicine, Icahn School of Medicine at Mount Sinai, New York, New York, USA, mountsinai.org; ^2^ Tisch Cancer Institute, Icahn School of Medicine at Mount Sinai, New York, New York, USA, mountsinai.org

## Abstract

Double minutes (dmins), a form of extrachromosomal DNA (ecDNA), represent a rare cytogenomic event in myeloid neoplasms and are most commonly associated with amplification of oncogenes such as *MYC* or *KMT2A*. Dmins derived from the 11q24 region that exclude *KMT2A* are exceedingly uncommon, and their pathogenic significance remains poorly understood. We report a 74‐year‐old female initially diagnosed with myelodysplastic syndrome (MDS) with isolated del(5) (q13q33) and mutations in *TP53* and *SF3B1*. After eight years and treatment with lenalidomide with excellent clinical response, she developed progressive cytopenias and transformation to acute myeloid leukemia, myelodysplasia‐related (AML‐MR). Cytogenomic analysis at the time of leukemic transformation revealed del(5) (q13q33) in all 20 metaphase cells analyzed and loss of the *EGR1* (5q31.2) gene in 94% of interphase nuclei by fluorescence in situ hybridization (FISH). Notably, 18 of 20 metaphase cells also harbored dmins, ranging from 2 to 22 copies per cell. Array‐based comparative genomic hybridization and single nucleotide polymorphism array (array‐CGH + SNP) identified a 5.57 Mb amplification of chromosome 11q24.2–q25 encompassing at least 40 genes, including *FLI1* and *ETS1* but excluding *KMT2A*. Metaphase FISH confirmed localization of the amplified 11q24 segment within the dmins, and immunohistochemistry demonstrated nuclear FLI1 expression in myeloblasts. The patient was treated with combination azacitidine and venetoclax and an investigational immunotherapy within a clinical trial. This case represents the third reported instance of dmins derived from the 11q24 region involving *FLI1* and *ETS1* and the first identified in the context of AML evolved from del(5q) MDS. Dmins in myeloid neoplasms have been linked to genomic instability, clonal evolution, and therapeutic resistance. Amplification and expression of *FLI1* in blasts, a hematopoietic transcription factor implicated in leukemogenesis and poor prognosis in AML, suggest a potential pathogenic role for 11q24‐derived dmins in disease progression. Our findings expand the spectrum of dmin‐associated oncogenic amplifications in myeloid neoplasms and highlight *FLI1* and *ETS1* as recurrent targets of 11q24‐derived ecDNA amplification. Recognition of such rare events underscores the importance of integrative cytogenomic profiling for uncovering novel mechanisms of leukemic transformation and potential therapeutic targets.

## 1. Introduction

Myelodysplastic syndromes (MDSs) are a group of clonal hematopoietic stem cell diseases characterized by cytopenia(s), dysplasia in one or more lineages, ineffective hematopoiesis, and recurrent genetic abnormalities. Interstitial deletion of the long arm of chromosome 5, del(5q), represents one of the most prevalent cytogenetic abnormalities in MDS, present in approximately 10%–15% of patients. The clinical course of MDS is often complicated by an increased risk of progression to bone marrow failure or to acute myeloid leukemia (AML). Progression to AML occurs in approximately 30% of overall MDS cases within a few months to a few years, whereas patients with MDS and isolated del(5q) have a markedly lower risk, with leukemic transformation rates below 10% [1]. While the genetic basis underlying the progression from MDS to AML is not yet fully elucidated, current evidence suggests a multifactorial process involving acquired mutations, clonal evolution, and genomic abnormalities.

Double minutes (dmins), also known as extrachromosomal DNA (ecDNA), are rare but recurrent chromosomal abnormality that contributes to the development of *de novo* AML as well as progression to AML from MDS [2]. *MYC* and *KMT2A* are the most frequently amplified genes in dmins in AML and MDS, which have been linked to poor prognosis and shorter survival, although their direct prognostic impact remains controversial [3]. Here, we report a case of AML evolved from MDS with isolated del(5q), characterized by dmins containing a segmental amplification of chromosome band 11q24.3 involving multiple genes but excluding *KMT2A*.

## 2. Case Presentation

**FIGURE 1:**
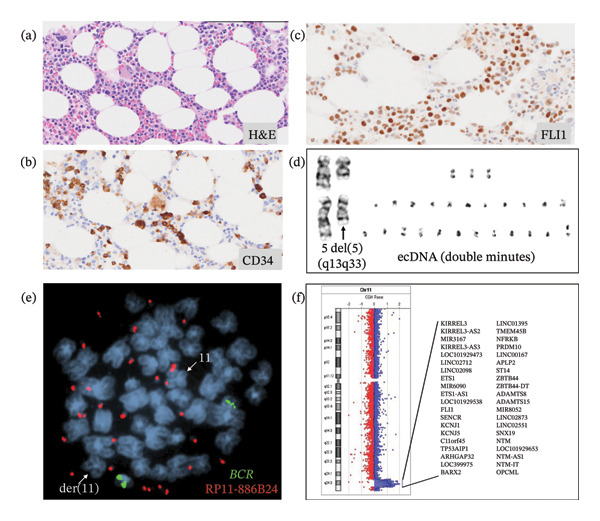
Histology, cytogenetics, FISH, and microarray findings of May‐2025 bone marrow biopsy. (a) H&E‐stained bone marrow core biopsy showing small and hypolobated megakaryocytes and hypolobated, hypogranular granulocytes in the background of trilineage hematopoiesis. (b) CD34 immunostain highlights immature blasts, comprising 20%–25% of the total cells. (c) FLI1 immunostain showing nuclear staining in a subset of the cells, indicating FLI1 protein expression in blasts. (d) Cytogenetic finding, showing interstitial deletion of 5q [(5) (q13q33)] and 22 double minutes (ecDNA) in one metaphase. (e) Metaphase FISH with a *BCR* locus specific probe and a bacterial artificial chromosome (BAC) FISH (RP11‐886B24) probe showing amplification of 11q24 genes within dmins. Only one chromosome 11 has 11q24 genes (arrow), indicating that one of the two chromosome 11 is missing 11q24 genes. Der(11): derivative chromosome 11. (f) Microarray on chromosome 11 showing loss of genes within 11q24 locus including *ETS1* and *FLI1*, but not including *KMT2A*(11q23).

A 74‐year‐old female with a history of hypertension and Hashimoto thyroiditis was diagnosed in 2015 with del(5q) MDS in the setting of progressive macrocytic anemia and mild neutropenia. Complete blood counts and differentials at the time of initial diagnosis are shown in Table 1. Initial bone marrow evaluation revealed a hypocellular marrow with myeloid dysplasia, loss of *EGR1* [del(5q)] in 15% of cells by fluorescence in situ hybridization (FISH), and an *SF3B1* mutation with a variant allele fraction (VAF) of 11.5%. She was initially monitored with blood counts every 2‐3 months, but in 2018, lenalidomide (10 mg daily) was initiated for progressive symptomatic anemia. This led to a transient decline in hemoglobin, reaching a nadir of 7.7 g/dL, but after dose adjustment to 5 mg daily, her hemoglobin improved to 13.3 g/dL. This hematologic response was sustained until late 2022, when she developed progressive anemia, new‐onset grade 1 neutropenia, and thrombocytopenia despite lenalidomide dose holds and schedule modifications. Annual bone marrow evaluations continued to show hypocellularity and megakaryocytic dysplasia, along with the emergence and expansion of a *TP53*R248W clone, with VAFs of 5.7% in 2020, 7.5% in 2021, 9.5% in 2023, and 22.5% in 2024. In early 2025, she developed worsening cytopenias without circulating blasts. Peripheral blood findings are shown in Table 1. Bone marrow biopsy at that time revealed 23% myeloblasts in a background of multilineage dysplasia. Notably, the *TP53* mutation was < 5%, while the *SF3B1* K700E mutation persisted (VAF 22.5%). Cytogenomic analysis revealed a deletion of del (5) (q13q33) in all metaphase cells analyzed by conventional cytogenetics and loss of *EGR1* (5q31.2) in 94% of interphase nuclei by FISH. For the first time, karyotypic evaluation identified 2–22 dmins in 18 of 20 (90%) metaphase cells analyzed (Table 2) (Figure 1). The final karyotype was designated as 46,XX,del (5) (q13q33)[2]/46,idem,2∼22dmin[18]. Array‐based comparative genomic hybridization and single nucleotide polymorphism array (array‐CGH + SNP) analysis identified a 5.57 Mb amplification of chromosome 11q24.2–q25 encompassing at least 40 genes, including *FLI1* and *ETS1* (Figure 1). Metaphase FISH using a *FLI1* probe confirmed localization of the amplified segment within the dmins. Immunohistochemical staining of the bone marrow demonstrated expression of FLI1 by myeloblasts (Figure 1). She was subsequently started on combination azacitidine and venetoclax and was randomized to receive an experimental immunotherapy clinical trial. Complete response was achieved after the second cycle with undetectable blasts on bone marrow flow cytometry. Follow‐up molecular testing revealed a significant reduction in *SF3B1* VAF, while *TP53* VAF showed a transient increase before declining. Cytogenomic testing showed a decreased clonal burden of the del(5q) population and no detectable dmins by either FISH or conventional cytogenetic analysis. The patient was scheduled to proceed with hematopoietic stem cell transplant at another institution.

**TABLE 1:** Peripheral blood findings at initial presentation and at the time of leukemic transformation.

	**2015**	**2025 May**

*Complete blood count*		
White blood cell	3.9	2 × 10^3^/µL
Red blood cell	3.5	2.91 × 10^6^/µL
Hemoglobin	12.2	9.2 g/dL
Hematocrit	35.6	27%
Mean corpuscular volume	102	92.8FL
Platelet	191	35 × 10^3^/µL

*Differential*		
Granulocytes	38.9	46%
Lymphocyte	45.6	40%
Monocyte	11.1	10%
Eosinophil	4.2	3%
Basophil	0.2	0%

**TABLE 2:** Chronological summary of bone marrow histology and cytogenetic changes.

Year	Blasts, morphology	FISH *EGR1* (%)	Cytogenetics del(5q) (%)	*SF3B1* VAF (%)	*TP53* VAF (%)	Number of dmins (% of cells)
2020	< 1% blasts, BM cellularity 5%, Mega and erythr dysplasia	20	20	11.5	5.7	0
2021	< 1% blasts, BM cellularity 5%–10%, Mega dysplasia	33	15	13.6	—	0
2022	< 1% blasts, BM cellularity 20%–30%, Mega dysplasia	47	45	8.8	7.3	0
2023	< 1% blasts, BM cellularity 20%–30%, Mega dysplasia	54	75	16.4	9.5	0
2024	2% blasts, BM cellularity 70%, Mega dysplasia	62	55	17.5	22.5	0
2025 May	19% blasts, BM cellularity 0%–60%, multilineage dysplasia	94	100	22.5	4.4	2 to 22 (90)
2025 June	4% blasts, BM cellularity 30%, Mega dysplasia	69	85	18.8	25.6	0
2025 July	1% blasts, BM cellularity 10%–20%, Mega dysplasia	32	Not Performed	5.9	7.9	0

*Note:* Blast percentage in the bone marrow core biopsy was measured by flow cytometry. Mega: megakaryocytic. Gran: granulocytic. Erythr: erythroid. FISH‐EGR1: percentage of cells with loss of EGR1 detected by FISH. Cytogenetics‐del(5q): the percentage of metaphases with del(5q) among 20 examined metaphases. SF3B1‐VAF: variant allele fraction (%) of SF3B1 mutation detected by next‐generation sequencing (NGS). TP53‐VAF: variant allele fraction (%) of TP53 mutation detected by NGS. Number of dmins: number of dmins detected per one metaphase during karyotyping.

## 3. Discussion

Dmins, first reported in 1962, are small, paired, extrachromosomal circular DNA elements that can be identified through conventional karyotypic analysis [4–6]. Dmins exhibit considerable variability in size and structural organization and frequently contain amplified oncogenes or genes conferring therapeutic resistance [7–9]. Their presence has been implicated in tumor initiation and clonal evolution [10–12]. Dmins have been identified in both solid and hematologic malignancies, with an overall frequency of ∼14% across cancers [13]. In hematologic neoplasms, *MYC* is the most commonly amplified oncogene in the form of ecDNA, present in approximately 0.1% of myeloid neoplasms, and *MYC*‐containing dmins are observed predominantly in myelodysplastic neoplasms where they frequently co‐occur with *TET2* mutations [14]. They are often associated with additional cytogenetic abnormalities, particularly complex karyotypes and recurrent chromosomal losses such as del(5q) (seen in ∼29%). Deletion of *NPM1* at 5q35 has also been strongly linked to dmin formation [15]. These findings may suggest that disease‐defining chromosomal lesions may predispose to dmin development during clonal evolution. Interestingly, several small series have shown that most *KMT2A*‐amplified cases also demonstrate MYC expression, implying that *KMT2A* may act upstream of *MYC* in a shared oncogenic pathway [3]. One case report described multiple copies of both *MYC* and *KMT2A* within chromosomal gain and ring chromosomes in a single AML case, further supporting the interconnection of the two genes [16].

While most reports of dmins in myeloid neoplasms frequently involve amplification of *MYC* (8q24) or *KMT2A* (11q23), particularly in MDS and AML‐MR, in contrast, dmins originating from the 11q24 region that exclude *KMT2A* are exceedingly rare. To our knowledge, this case represents only the third report of dmins with amplification restricted to the 11q24 region, involving genes other than *KMT2A*. Previously, only two such cases have been described, one in AML‐M2 [17] and another in refractory anemia with excess blasts [18]. Similar to previous reports, our case also demonstrated amplification of *FLI1* and *ETS1*. *FLI1* is a transcription factor with oncogenic potential that plays a pivotal role in hematopoietic development. It is essential for hematopoietic stem cell maintenance and for erythroid and megakaryocytic differentiation [10]. Dysregulated *FLI1* expression has been implicated in leukemogenesis, and its overexpression is associated with adverse prognosis in AML [19]. In our patient, immunohistochemistry demonstrated strong FLI1 expression in the bone marrow blasts. This region also harbored the *ETS1* gene, which is also considered an oncogene, with dysregulated expression reported in adult T‐cell leukemia, diffuse large B‐cell lymphoma, ovarian cancer, and prostate cancer [20]. In human hematopoietic progenitor cells, overexpression of ETS1 impairs erythroid differentiation and promotes commitment toward the megakaryocytic lineage [20].

In our unpublished institutional database (2017–2025), we identified 21 patients with extrachromosomal amplifications and homogeneously staining regions (HSRs), an abnormal chromosomal segment harboring gene amplification: 7 with dmins (all with MDS at diagnosis) and 14 with HSR (associated with AML, MPN, or ALL but not MDS). Of the 7 dmin cases, 5 involved *MYC*, 1 involved *DNMT2*, and the present case harbored amplification of an 11q24 segment.

Taken together, our case represents the third published instance of dmins derived from the 11q24 region, amplifying *FLI1* and *ETS1*, but notably excluding *KMT2A* localized at 11q23, the second most commonly amplified gene in dmins in AML. These findings highlight *FLI1* and *ETS1* as recurrent targets of dmin‐mediated amplification in myeloid neoplasms and suggest that extrachromosomal amplification of this region may play a pathogenic role, with potential implications for therapeutic resistance. Notably, the patient’s favorable response to immunotherapy suggests that immune‐based treatment strategies may, in some cases, mitigate the poor outcomes typically associated with dmins, and it is conceivable that the absence of high‐risk oncogene amplifications such as *KMT2A* or *MYC* may have contributed to this improved therapeutic responsiveness.

## Funding

This study was not supported by any sponsor or funder.

## Consent

No written consent has been obtained from the patients as there are no patient identifiable data included in this case report.

## Conflicts of Interest

The authors declare no conflicts of interest.

## Data Availability

Data sharing is not applicable to this article as no datasets were generated or analyzed during the current study.
